# 

**Published:** 1879-07

**Authors:** 


### Editorial.

### AMERICAN DENTAL ASSOCIATION.

The nineteenth annual meeting of the *American Dental Association* will be held at Niagara Falls, beginning on Tuesday, August 5th, at ten o'clock a. m., and continue four days.

The sessions will be held in the "Pavilion" in Prospect Park.

One admittance fee, (twenty-five cents) to the park, will suffice for the whole time of the meetings. The park and falls will be illuminated by the electric light every evening.

The International and Cataract Hotels, have reduced rates to three dollars per day, for those attending the Association. About half rates are provided to all the places of interest and attraction about the Falls.

All, upon arriving at the Falls, should call immediately upon Dr. G. L. Field, at the International Hotel, who will give all information as to details.

Arrangements have been made with the various railroads centering at Niagara, for special rates for those attending the meeting.

For securing these rates, application must be made to the following persons: Those who will pass over the "*New York Central*" or the "*Erie*," apply to Dr. Frank M. Oddell, No. 7 w. 38th st., New York City.

Those over the "*Wabash and Great Western*," to Dr. Geo. L. Field, Detroit, Mich.

Parties going from St. Louis and vicinity, apply to Dr. G. W. Spalding, St. Louis.

Those going from Cincinnati and vicinity, or through it, will obtain tickets of Dr. J. Taft. The round trip from Cincinnati will be ten dollars.

Those going this route please give notice as early as possible.

The above embodies the communications received from Drs. Geo. H. Cushing, Thos. Fillebrown and Geo. L. Field, officers of the Association.

The indications are that there will be a large number in attendance at the coming meeting. There are many inviting circumstances. Indeed, Niagara is always inviting.

### AMERICAN SOCIETY OF MICROSCOPISTS.

The second annual meeting of this Society will be held in the city of Buffalo on the 19th day of August next.

A general local Committee of Management, consisting of the Buffalo Microscopical Club and kindred societies of that city, has been appointed, which will take charge of all matters pertaining to the reception and proper accomodation and entertainment of the members of this important national convocation.

Everything is being done to promote the convenience, economy and comfort of the visiting body, and to render its session successful and auspicious, and its sojourn gratifying to all concerned.

Arrangements are being made with the various railroad authorities, which will enable members of the convention to secure passage to and from the meeting at reduced rates.

This will doubtless be an exceedingly interesting occasion to all in any wise engaged in microscopical science. Let all be present who can. Any desiring further information may address either Dr. H. R. Hopkins, President, or Mr. James W. Ward, Secretary, both of Buffalo, New York.

### VIRGINIA STATE DENTAL ASSOCIATION.

The next annual meeting of this Society will be held at Charlottsville, Tuesday, August 19th, 1879, at ten o'clock a. m.

All members of the profession, not only in Virginia, but in sister States, whether members of the Society or not, are cordially invited to be present at the meeting and take part in the discussions. It is the expressed wish of the officers that each member of the several committees will have a well digested report to present at that meeting.

There are committees upon the following subjects, viz.:

1. "Operative Dentistry," of which Dr. W. H. H. Thackston is Chairman.
2. "Mechanical Dentistry." Dr. H. C. Jones, Chairman.
3. "Education and Literature." Dr. W. E· Norris, Chairman.
4. "Pathology," Dr. H. M. Grant, Chairman.
5. "Physiology, Histology, Microscopy and Chemistry." E. R Perrow, Chairman.
6. "Dental Appliances." C. A. Mercer, Chairman.

This meeting will doubtless be an occasion of great interest and profit to those especially who may be present.

Dr. L. M. Cowarden, of Richmond, is Corresponding Secretary.

### WISCONSIN.

The ninth annual meeting of the *Wisconsin State Dental Society* will be held at Arion Hall, Milwaukee, July 15th, 1879, and continue three days. The following excellent programme is presented for the occasion:

1. President's Address.
2. Popular Education on the Teeth. A. Holbrook.
3. Alveolar Abscess: "Treatment by a Country Dentist." T. B. Fletcher.
4. Talk on Dental Irregularities. H. Enos.
5. The Origin and Early Development of the Teeth and Associate Parts. M. S. Dean.
6. New Instruments, Appliances, Medicines, etc.
7. "New Departure." R. S. Wells.
8. Question Box.
9. Nervous Patients. W. L. Lewis.
10. State Law to Regulate the Practice of Dentistry.

Volunteer essays are solicited, especially upon the greatly neglected subject of Mechanical Dentistry. Arrangements have been made for holding an extended series of clinics to which volunteer operators are especially invited.

All will be most cordially received and welcomed. Arrangements have been made with hotels and railroads for very low rates. Let everybody go.

### NEW JERSEY.

The ninth annual meeting of the *New Jersey State Dental Society* will be held in Seaside Chapel, Long Branch, Wednesday, July 16th, at ten o'clock, and continue three days.

The following topics are upon the programme for discussions, viz.:

"The Practice of Dentistry." J. Hayhurst.

"Little Things." T. B. Wech.

"Our Mission." J. G. Palmer.

"The Elevation of the Dental Profession to Its Proper Standard." F. W. Levy.

"Dentistry As It Was, and Now Is." J. Chadsey.

"The Use and Abuse of Mercury." J. W. Scarborough.

"Vulcanite vs. Metal Plates." E. Chew.

Clinics on Thursday and Friday mornings.

Members are especially urged to come prepared to report incidents of office practice. A portion of each day will be devoted to that order. A general and cordial invitation is extended to the profession.

The Board of Examiners will convene at two o'clock p. m., Tuesday, July 14th. Candidates for license to practice according to law, must be on hand promptly at that time and bring with them, for deposit, a specimen of the mechanical work, and be also prepared to fill a tooth with gold in the presence of the Examiners.

### CONTENTS.

COMMUNICATIONS.

### *THE DENTAL REGISTER,*

A MONTHLY MAGAZINE DEVOTED TO THE INTERESTS OF

### THE DENTAL PROFESSION.

**Terms Reduced to $2 50 per Year. Single Copies 25c.**

*EDITED BY PROF. J. TAFT, D. D. S.,*

AND PUBLISHED BY

SPENCER & CROCKER, Ohio Dental Depot.

The volume commences in January and closes in December.

The REGISTER being issued monthly is always fresh, therefore Indispensable to the practitioner who desires to keep posted up with the new inventions and improvements which are daily developed. Neither expense nor effort will be spared to give value and variety to its contents. Articles will be illustrated with well executed engravings whenever they will add to the interest or assist to explanation.

Its extensive circulation and frequency of issue make it one of the BEST DENTAL ADVERTISING MEDIUMS in the United States.

All subscrptions must begin with January or July.

Local or special notices, five lines of less, will be inserted for $3.00 each insertion; over five lines, 50 cts. per line.

All advertising bills payable in advance, or at furthest, after the issue of the first number containing the advertisement. All transient advertisements to be paid for in advance.

☞CONTRIBUTIONS SOLICITED.

Exclusively Editorial Communications should be addressed to Editor.

The latest number of the REGISTER may be ordered with or without other goods at any time, and all letters should be addressed to

**SPENCER & CROCKER, Ohio Dental Depot, Cincinnati, O.**

